# Patterns of the radiation properties for Peano antennas

**DOI:** 10.1038/s41598-023-35185-6

**Published:** 2023-05-24

**Authors:** René Pérez-Moroyoqui, Suemi Rodríguez-Romo, Oscar Ibáñez-Orozco

**Affiliations:** Centro de Investigaciones Teóricas, Facultad de Estudios Superiores Cuautitlán, Universidad Nacional Autónoma de México, Cuautitlan, Izcalli Mexico

**Keywords:** Engineering, Mathematics and computing

## Abstract

This paper studies metallic microstrip antennas with air as a substrate in the UHF band, patterned after space-filling, self-avoiding, and self-similar (FASS) Peano curves. Our novel study is based on context-free grammar and genetic programming as computing tools to unravel the role of geometry on both; the Voltage Standing Wave Ratio (VSWR$<2$) and frequency resonance patterns for Peano antennas. We use in our approach the numeric method of moments (MoM) implemented in Matlab 2021a to solve the corresponding Maxwell equations. Novel equations for the patterns of both features (resonance frequencies and frequencies such that the VSWR$<2$) are provided as functions of the characteristic length *L*. Antennas spanning a $L\times L$ area ($L\le 0.1\,m$), feeding points set at seven places, and three widths of the metallic strip are introduced as instances of our approach. Finally, a Python 3.7 application is constructed to facilitate the extension and use of our results.

## Introduction

New IoT technologies require efficient information transmission through multiband, compact antennas; novel applications test different, uncommon antenna designs. On the other hand, compact antennas are central to some wireless networks and personal communication systems applications. Fractal geometry helps design small multiband antennas, arrays, and high-directive elements in antenna engineering. Furthermore, some fractals present copies of the whole structure at small scales; this attribute is called self-similarity, a feature that benefits the design of multiband antennas^1–3^.

There are many problems in wireless communications, including, for instance: (1) Increasing the effective range of an antenna by increasing its surface area. (2) Improving signal reception in locations where the signal is weak. (3) Reducing the antenna size can be essential in portable devices. (4) Reducing the amount of power needed for transmission and reception. (5) Reducing the amount of interference caused by other nearby devices. Fractal antennas constitute a way to address these issues. The evolution of wireless communication technology has increased the demand for wideband, multiband, small, and high-efficiency antennas. These antennas with such features are used due to their simple designs and ease of integration. Many researchers are focused on designing antennas that obey the abovementioned characteristics. Therefore, several techniques are used to overcome the problem of considerable size and keeping the wideband or multiband behavior.

Great scientists contributed to the study of fractal geometry: Sierpinski, Hilbert, Minkowski, von Koch, and Peano et al. Several fractal curves have been used in the literature as geometries for antennas^4^, for instance, Minkowski, Koch, Moore, Hilbert, Peano, Sierpinski, and Dragon antennas^5–8^. Hilbert curve, proposed by Hilbert in 1891^9^, is one of the famous space-filling, self-avoiding, and self-similar (FASS) curves studied to design antennas. FASS antennas provide compact structures for different geometries of interest. Intriguing features for Hilbert antennas have been found so far^6,10–17^. Additionally, Giuseppe Peano made a fundamental contribution to fractals by defining (in 1890) a continuous curve (named after him) with infinite singularities that fills an area^1^. Peano curve has inspired antenna engineers to design small antennas, high-directive elements, arrays, and frequency selective surfaces^18–27^. Additional studies in this line of thinking have been reported elsewhere^20,28–32^.

This paper systematically studies deterministic fractal microstrip metallic and FASS antennas, named Peano antennas; we analyze two electrical features, resonance frequency and VSWR$<2$ in the UHF band as the production rule iteration increases for four (seven due to symmetry) different feeding points, spanned areas smaller than 0.1 m $\times$ 0.1 m, and three different microstrip widths. Besides, we chose air as the Peano antenna substrate to focus only on the role of the antenna geometry (production rule, feeding points positions, strip widths, and spanned area) over the resonance frequency and frequencies such that VSWR$<2$. FASS antennas are used in telecommunications, among others, and are an object of current interest. On the other hand, classical antennas use simple curves for their design; they have been widely studied and used for everyday purposes, such as dipoles, monopoles, and logarithmic antennas. The radiation properties of classical antennas are well-known; easily calculated using commercial software such as COMSOL Multiphysics, Ansys HFSS, FEKO, HFWorks, Sonnet Software, WIPL-D Pro, Empire XPU, Remcom Xfdtd, AWR Microwave Office, and CST MICROWAVE STUDIO.

Multiband capacity and compact structures are needed for today’s highly demanding applications; the same communication device demands connecting multiple services. Here, we address the UHF frequency band (300 MHz–3000 MHz) because of the potential commercial applications; however, our approach can deal with any other frequency band of the electromagnetic spectrum. The UHF applications include industrial, scientific, and medical (ISM) radio bands (433.92 MHz, 915 MHz, 2.45 GHz), GPS tracking systems (1575.42 MHz, 1227.60 MHz, 1381.05 MHz, 1176.45 MHz), space-based satellite navigation systems as Galileo (1575.42 MHz, 1191.795 MHz, 1176.45 MHz, 1207.14 MHz, 1278.75 MHz) and GLONASS (1602.0MHZ), L-band uplink/downlink for the Low Earth Orbit Iridium Satellite Constellation (1618–1626.5 MHz), cellular communications (870–960 MHz), WiFi 802.11b, 802.11g and 802.11n (2400 MHz), and radio-frequency identification or RFID (868–956 MHz), (2400-2483.5 MHz) used as radio astronomical research (1660.5–1668.4 MHz), among others.

The research by Zhu et al.^6^ of Peano antennas inspired us. In this paper, the authors analyze the radiation properties of several Peano antennas. They study iterations *i* = 1, 2, 3, 4 of the Peano fractal, one resonance frequency for each iteration, *L* = 70 mm, and one off-center feed point per iteration. The geometry of the Peano antennas proposed by the authors is those with approximately 50 ohms input impedance for n = 1, 2, 4, and 75 ohms for n = 3 at its fundamental resonant frequency. They also analyzed the different bandwidths around the central frequency, such as VSWR < 2 per iteration. In this framework, Zhu et al. show how the Copper Peano antenna, at the fundamental resonance frequency, reduces radiation efficiency from 97.06 to 72.25% and 17.5% with the iteration order.

In this paper, the variable *F* represents the frequency and can take any value within the UHF frequency band. Namely, *F* can represent any specific frequency within that range (300 MHz to 3 GHz). For example, *F* could be 500 MHz, 1.2 GHz, or 2.7 GHz, as long as it is within the limits of the UHF band. The variable *L* describes the length of the side that encloses the Peano curve. As the amount of iterations of the Peano curve increases, the total length of the curve increases, but the square that contains it continues to have sides of length *L* chosen arbitrarily in the range from 0.01 m to 0.1 m.

The variable *S* is the total length of the Peano curve, the sum of all segment lengths comprising the curve; each iteration *S* increases. *S* is a function of the number of iterations and the initial size of the segments that constitute it. We choose a set of feeding points in different arbitrary positions as fractions of the *S* length to study the patterns for VSWR$<2$ and resonance frequencies.

Let us stress that our approach involves computational simulations of a significant number of antennas to understand the behavior of the two electrical design features chosen; the resonance frequency and the frequencies such that VSWR$<2$. The fundamental idea behind our proposal is that the Peano analysis already reported in the literature constitutes few particular cases. A systematic study is necessary; we intend to contribute to filling this gap here. The frequency range to perform our research includes the UHF band, seven different feeding points, three widths of the strip *d*, and *L* in the range from 0.01 m to 0.1 m as instances of our approach that we assume are representative enough.

Thus, we differentiate from the research performed so far since, in our study, we span the behavior of the two antenna electrical features into a range (UHF) of frequencies, feeding point positions, *L* values (therefore, the spanned area $L\times L$) of the antenna, and the widths of the strip. We intend to isolate the effect of these variables on the two electrical features chosen as a design metric since the air as a substrate guarantees the radiation efficiency of (around) 100% for our antennas. Another substrate should be elected to build real antennas and analyze radiation efficiencies.

Since we chose air as a substrate, our Peano antenna is ideal. Introducing a different, more realistic substrate would change this; let us take, for example, a Peano iteration two antenna with Teflon as a substrate. This antenna produces radiation efficiencies from 5% (0.75 GHz ) to 73% (2.85 GHz) as a function of the frequency of the UHF band. For Peano, iteration 3, with Teflon as a substrate, we calculate the efficiencies as a function of the frequency. In this case, we obtain values from almost zero at around 1.3 GHz to almost 30% at around 2.85 GHz. It is well known^33^ that there are two categories to produce and study miniature antennas; the topological-based and the material-based; we focus on the first one in this paper, assuming that this splitting holds for our cases. Namely, we think that the effect of the geometry, *L*, the width of the strip, the frequency range spanned, and the feeding points can be factorized from the nature of the substrate about pattern formation in the properties of the Peano antennas.

Furthermore, instead of studying only one resonant frequency^6^, the Peano antenna radiation properties are scanned along with the UHF band. We consider that our approach contributes to the study of the properties of Peano antennas, a particular case of FASS antennas. Besides the standard MoM method to solve Maxwell equations, we introduce novel Artificial Intelligence tools like genetic programming by GPlearn. Overall the computational tools used here have allowed us to generate equations that relate the antenna size *L* with *F*, being *F* either the resonance frequency or the central frequency such that $VSWR <2$. Besides, we obtain the bandwidths around the central frequencies such that VSWR$<2$. Thus, we provide a deeper understanding of Peano antenna features than the results already reported in the literature.

The variables to study will be the electrical resonance of the antenna and the VSWR. First, energy transfer between the antenna and the transmission line (such as a coaxial cable) is more efficient when an antenna is in resonance because the antenna’s impedance and the transmission line’s impedance can be more easily matched, which minimizes reflections and energy losses. A low VSWR guarantees that most of the energy is transmitted through the antenna instead of being reflected in the transmission line, resulting in a better quality of the transmitted or received signal.

We provide equations for two conditions of a suitable Peano antenna design; (a) the resonance frequencies and (b) the frequencies such that VSWR$<2$ as functions of the length *L*. As far as we know, this is the first report of such equations (or any other mathematical model) on this subject. If VSWR equals 2, the incident power reflected is 11.11% of $\Gamma ^{2}$ ( where $\Gamma$ is the reflection coefficient) at the antenna feeding point. This value is a sensible criterion for producing antennas suitable for use in several different technological fields^4^.

**Figure 1:**
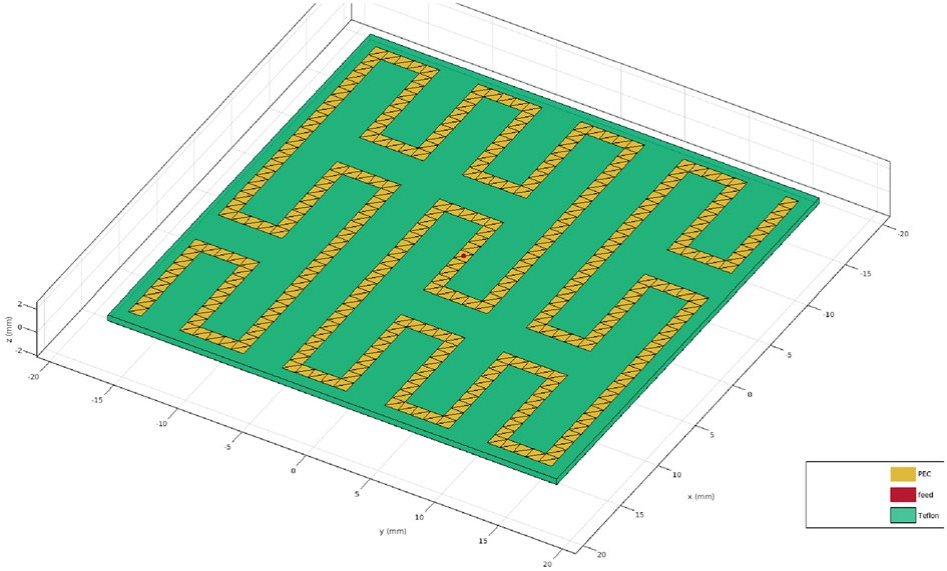
Peano antenna iteration 2, the line in yellow represents the perfect conductor (for instance, a metal), the red point is the feeding point of the antenna, and the green area is the substrate (Teflon).

The Peano microstrip antenna can be fabricated using photolithography and etching techniques on a dielectric substrate. Precision in the manufacturing process is essential to ensure that the patch geometry and antenna characteristics are consistent with the design. Figure 1 shows a Peano 2 antenna with a Teflon substrate. An antenna’s substrate affects its efficiency and performance in size, bandwidth, and radiation efficiency. When designing an antenna, selecting a substrate with the appropriate characteristics is essential to achieve an optimal balance between size, weight, efficiency, and performance. In this work, the antenna’s efficiency ($\eta$) is almost perfect when using air as the substrate. However, when other substrates are simulated, the antenna’s performance may drop to 0.7 in some instances. Please note that this study does not cover radiation efficiency as there are numerous substrate and configuration options to consider.

An application in Python 3.7 is provided; it is available on GitHub to access the full extent of the results and examples reported here and produce additional ones that may be useful for the researchers’ community^34^. This application works finding: (a) the resonance frequencies and b) frequencies such that VSWR<2) to produce Peano antennas spanning a square area smaller than 0.01m$^2$, in the UHF band, with four (seven by symmetry) feeding point positions and three microstrip widths.

This paper is organized as follows. The following section introduces the model and gives an insight into the simulation details. Then, in Section 3, we present results, first (Subsection A) for the resonance frequency analysis and second for the VSWR$< 2$ (Subsection B) analysis. In Section 4 we summarize and conclude.

**Figure 2:**
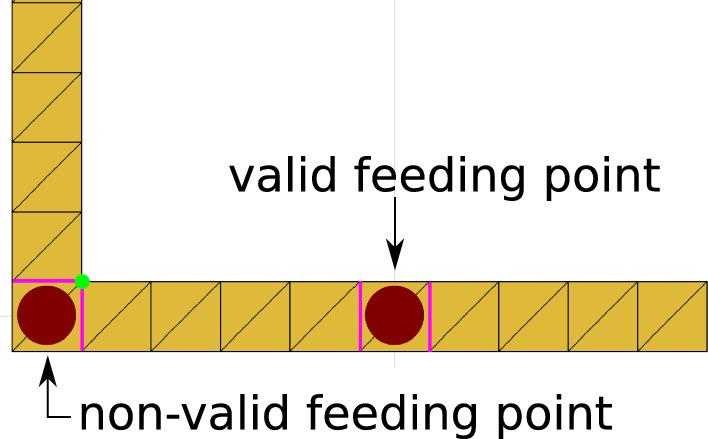
Zoom to a segment of the Peano antenna close to a corner. The mesh used by the method of moments (MoM) is shown. In the valid feeding point the [magenta] edges do not touch. In the non-valid feeding point the [magenta] edges touch in the euclidean point [green].

## Numerical simulations

We choose metal to build the microstrip Peano antennas and air as the substrate to simplify the simulations and focus on the influence of the topology of the antenna, namely, the production rules iteration of the fractal Peano antenna, the feeding point positions, the spanned area by the geometry, and the microstrip Peano antenna width over the performance. Our simulations are conducted from 290 MHz to 3100 MHz; this frequency range contains the UHF frequency band and avoids discontinuities and other numerical problems in the UHF band boundaries. In addition, we use a 50 ohms resistance in the feeding line throughout this paper.

Here, the method of moments (MoM)^35^ is applied to solve the Maxwell equations on the meshed metallic microstrip Peano antenna with air as substrate using the 2021a) MATLAB antennas toolkit^36^. We mesh the Peano antenna with square segments $d\times d$, where *d* is set to 0.25 mm, 0.5 mm, and 1 mm; see Fig. 2. Figure 2 shows two feeding points; the first is a valid feeding point for the numerical computation since both (magenta) edges do not touch. The second is a not-valid feeding point since the (magenta) edges coincide in the euclidean (green) point, producing an electrical short circuit. The number of these segments is proportional to *S*; see Table 1. Please note that the number of corners in the Peano curve increases with the production rule *i*. Corners need a particular treatment in the MoM numeric; the feeding points cannot be placed there because the computing does not produce valid results.

**Table 1:** Minimum and maximum number of squares segments in the Peano antenna for different iterations of the production rule.

Antenna	d = 0.25mm		d = 0.5mm		d = 1.0mm	
	Min	Max	Min	Max	Min	Max
Peano iteation 1	17	1617	73	809	33	401
Peano iteation 2	341	4001	241	2001	161	1041
Peano iteation 3	1457	11649	1457	5825	1457	2913

These segments have a size of $d\times d$, being *d* the width of the microstrip.

**Figure 3:**
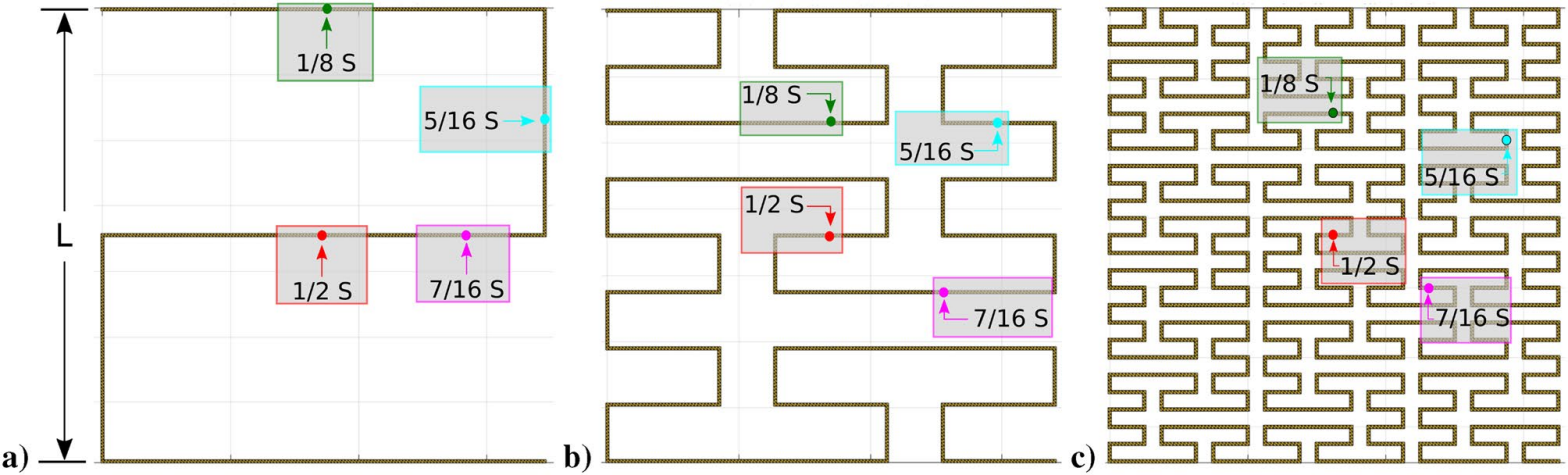
Feeding points 1/2*S* (red), 5/16*S* (cyan), 7/16*S* (magenta) and 1/8*S* (green). (**a**) Peano antenna, $i=1$ (Peano_1), (**b**) Peano antenna *i* = 2 (Peano_2), and (**c**) Peano antenna, $i=3$ (Peano_3).

We study Peano antennas with the feeding point placed at 1/8, 5/16, 7/16, and 1/2 times *S* (see Fig. 3). These Peano antennas are symmetrical under a turn counterclockwise of 180$^{\circ }$. Please note that this Peano antenna symmetry allows us to extend our results to antennas with feeding points positions at 9/16 (equal to 7/16) times *S*, 11/16 (equal to 5/16) times *S*, and 7/8 (equal to 1/8) times *S*. The Peano antennas with feeding points in (1/8*S*, 5/16*S*, 7/16*S*, 1/2 *S*,9/16 *S*,11/16, *S*,7/8 *S*) are the only feeding-point instances studied in this paper. Besides, we use a lateral size *L* in the range of (0 m, 0.1 m) and production rule iteration (*i* = 1,2,3).

The symbolic regressor GPlearn^37^ identifies the underlying mathematical expressions in the data obtained from both simulations; resonance frequencies and the frequencies such that VSWR<2. At first, perform simulations for an iteration *i* of the production rules, size *L*, allowed feeding point, and microstrip width value. Next, we ran a batch of 1000 genetic algorithms on the data with randomly chosen configuration parameters, number of generations, and algorithm initial population. We report the resonance frequency and frequencies equations such that VSWR<2 as functions of the antenna size *L* that defines the spanned $L\times L$ area.

The Peano antenna simulation results, namely, the resistance, reactance, and VSWR simulations, produce a database saved for further analysis.

Our method constructs a Peano antenna geometry using an L-system geometry generator that sets an antenna width *d*, a feeding point, and an *L* for the spanned area $L\times L$. Then we calculate the electromagnetic features for the antennas using MoM in MATLAB 2021a^36^. This procedure is repeated until generating a database of 996 antenna simulations. The MoM computing is the only part of the algorithm that uses MatLab; the remaining computing is homemade software coded in Python 3.7. The electromagnetic features produced by our simulations could be obtained by any other (commercial) software or computed directly from the Maxwell equations in Python 3.7. We use the Matlab 2021a antennas toolkit for expediency.

In the resonance frequency module of our method, we extract from the original basis (with 996 elements) one basis of the form (*F*, *L*) and apply genetic programming using GPlearn^37^ to find the best and simplest equation $L=f(F)$ to fit the data; we found this to be $L=aF^{-1}+b$. Then, we obtain the best *a* and *b* by the minimum least square. In the following module, from the original basis (with 996 elements), we get all the antenna frequencies such that VSWR < 2; and produce another (*F*, *L*) basis. Again, applying GPlearn^37^, we verify that $L=aF^{-1}+b$ is the type of equation that fits best this new second database. Here *a* and *b* are obtained by minimum least square, as was done in the resonance frequency module^34^.

In the solver module, we implement two types of problems; a) set *F* to find the best *L* for any Peano_i antenna that resonates and has a VSWR < 2, and b) set *L* and find the best resonance frequency such that VSWR < 2. Finally, the remaining Peano_i properties are calculated^34^.

## Results

This Section presents the patterns in resonance frequencies and frequencies such that VSWR $< 2$. First, we introduce novel equations that characterize the resonance frequency and frequencies such that VSWR<2 as leading rules to produce small Peano antennas (covering an area smaller than 0.01m$^2$) with adequate performance. We start by introducing the resonance frequency analysis for *i* = 1, 2, 3. Then, we study the frequencies where VSWR<2 for *i* = 1, 2, 3.

This paper’s most essential results are the equations obtained using GPlearn as a symbolic regressor for the resonance frequencies and frequencies such that VSWR<2 as functions of *L*; both equations are of the form $L=aF^{-1}+b$. Here *a* and *b* are parameters to be obtained using least-square fitting. *F* is the resonance frequency for the first case and the central frequency such that VSWR < 2 in the second case. We also provide the second-degree polynomial coefficients that relate the so-called central frequency (the mean value) such that VSWR< 2 with the bandwidth around this as a percentage of the central frequency. *F* is always in MHz and *L* in meters. These curves are valid only in the UHF band; we should test other band frequencies in the near future.

Our simulations produce many numerical results that we illustrate in this paper. Since these simulation results plus additional numeric can be obtained through the application Peano_antennas^34^, we consider that the examples presented here and this application available in GitHub represent well all the possibilities of our approach. Therefore, in the following Subsections, we introduce some of our simulation results via examples.

**Figure 4:**
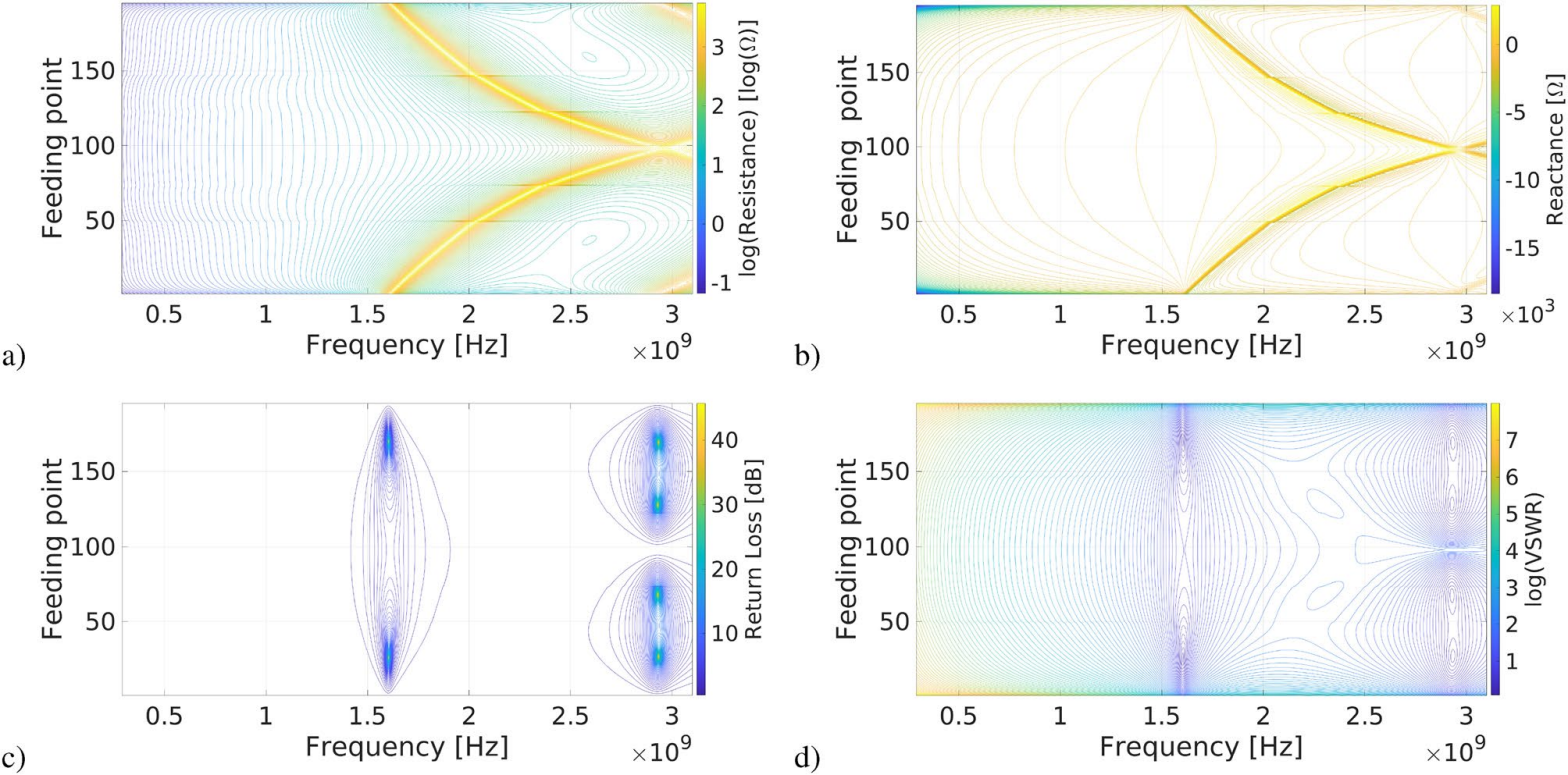
Electric properties of a Peano_1 antenna along the structure, *L* = 0.0225m, *d* = 0.5mm and 195 feeding points; (**a**) logarithm of the resistance, (**b**) reactance, (**c**) return loss (**d**) logarithm of the VSWR.

**Figure 5:**
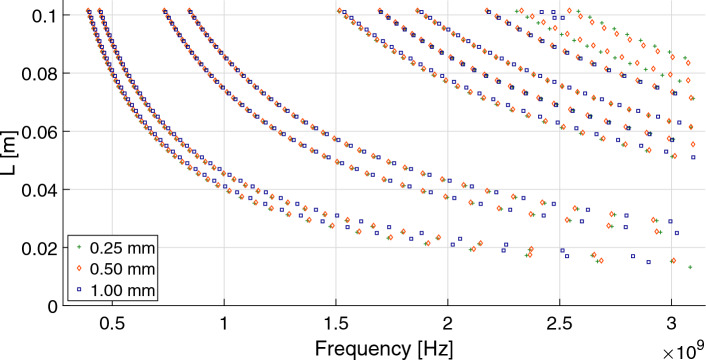
Simulated resonance frequency for the Peano antennas, *i* = 1 (Peano_1) with feeding point placed at 0.125 (1/8) times the length *S*, $L<0.10$ m, and antenna width of 0.25 mm, 0.5 mm, and 1.0 mm.

**Figure 6:**
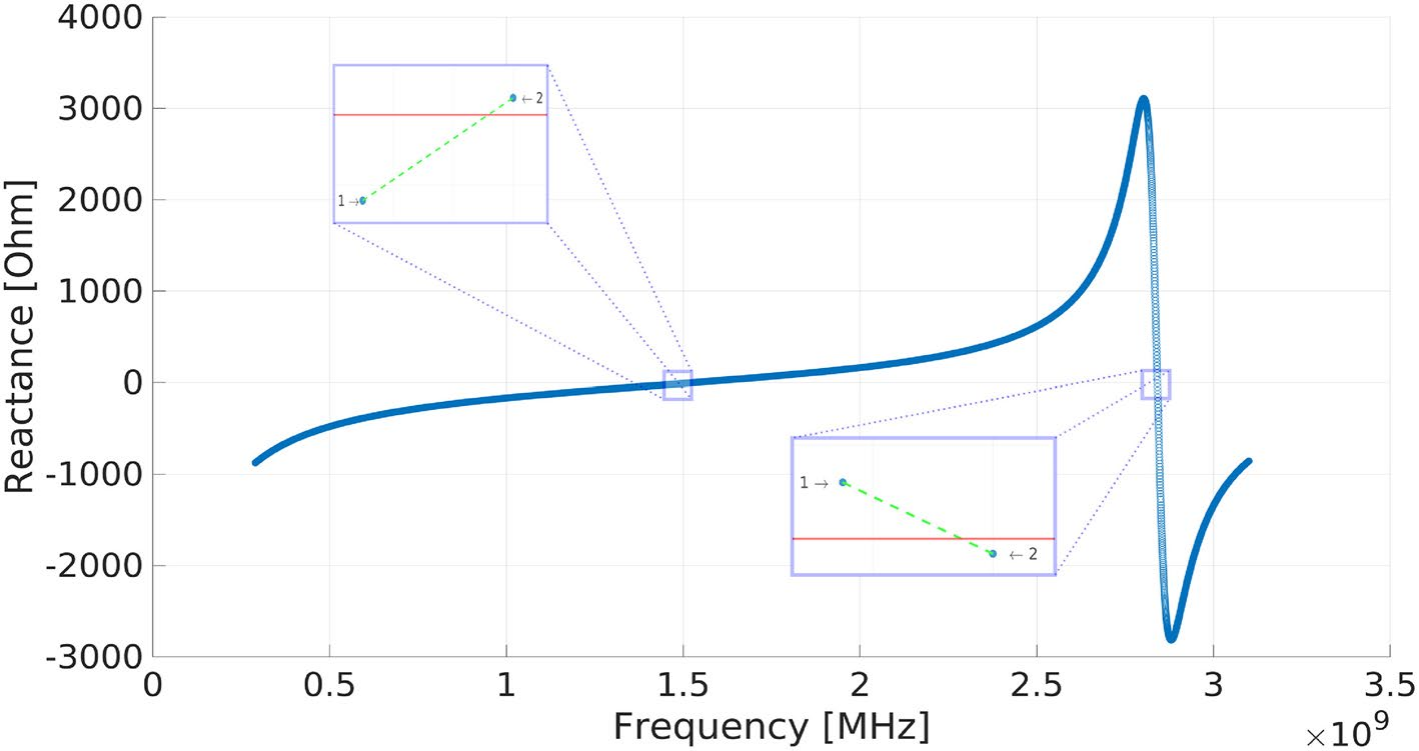
Calculated reactance of a Peano antenna at the feeding point 1/2*S*. The zero crossing points are shown.

### Peano antennas: resonance frequency equations

We simulate the Peano antenna resonance frequencies, sweeping frequency from 290 MHz, with a frequency increment of 0.25 MHz (for *i* = 1, 2) and 1MHz (for *i* = 3), up to 3100 MHz. We obtain 11241 impedance data (for *i* = 1, 2) and 2811 impedance data (for *i* = 3). Then, we search for the zero crossings of the reactance; thus, we calculate the resonance frequencies. For the Peano antennas iteration 3, we perform calculations by a frequency step of 1MHz to keep a sensible balance between the computation time used for the simulations and the antenna complexity. We study the resonance frequency equations for Peano antennas generated by production rule iteration *i* = 1, 2, 3.

Figure 6 shows how the electrical resonance of the antenna is calculated. The reactance property obtained in the feeding point is shown throughout the UHF band, and in two points, the resonance has a zero crossing. This figure shows an approach to these points, as seen in each value calculated before and after the crossing. For the calculation of the resonance, an equation is created between points 1 and 2 of the property and is resolved to obtain the most exact point of the zero crossing.

#### Peano_1

We show our results for a Peano antenna (production iteration rule equal to one); see Fig. 3a). In order to give an idea of the complexity of our simulations, we introduce our study with two examples.

##### Example 1

Let us consider *L* as 0.0225 m, the antenna width of 0.5mm, and all the possible feeding points, excluding corners, in total 195, throughout the antenna length *S*. The properties of these antennas in the UHF band can be seen in Fig. 4. Please, note that in Fig. 4a) we use the logarithm of the resistance and in Fig. 4d) the logarithm of the VSWR. Figure 4b) shows the antenna reactance; note that if this is zero, resonance occurs. The VSWR depends on the line’s resistance (in this case, 50 ohms) and the resistance at the feeding point. It is clear from Fig. 4 that there are patterns in the electrical radiation properties of the Peano antennas that potentially can be described by equations; obtaining some of them is the goal of this paper. We reach this goal and present our results below.

##### Example 2

Figure 5 shows the calculated resonance frequencies within the UHF band for Peano_1 antennas, $L<0.10$ m with feeding point placed at 0.125 (1/8) times the length *S*, and *d*=0.25 mm, *d*=0.5 mm, *d*=1.0 mm. Recall that the resonant frequencies depend on the feeding point’s position, the geometry, and the microstrip antenna widths. Patterns, easily described by equations, appear in Fig. 5. We split this Figure into three cases, each for a particular *d* value: (a) 0.25 mm, (b) 0.5 mm (c) 1.0 mm. The resonance frequencies are reported for the UHF band (to be more precise, $300\,{\text{MHz}}<F<3000\,{\text{MHz}}$) for different *L* ($L<0.1$ m) values, and the feeding point is placed at 0.125 times *S*. We apply GPlearn, dynamic programming, to obtain the possible functions to describe the curves shown in Fig. 5.

**Figure 7:**
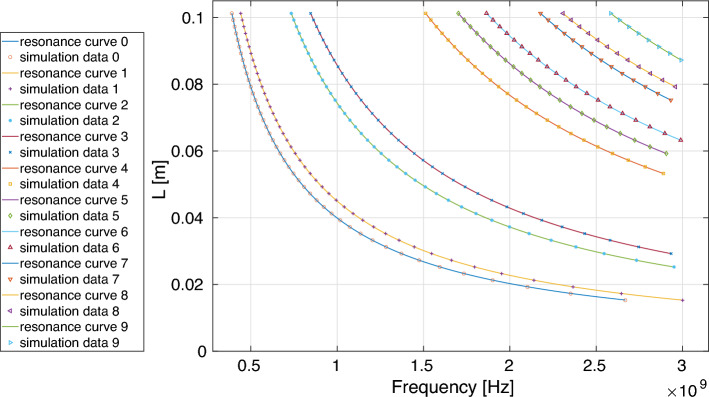
Resonance frequency *F* as a function of the length *L* for Peano_1 antennas with feeding point placed at 0.125 (1/8) of *S* for antenna width 0.25mm.

The best possible functions that fit these curves are $L=aF^{-1}+b$. The parameters *a* and *b* for all the curves shown in Fig. 5 are given in Table 2. In this Table, besides the corresponding parameters, *a* and *b* for each curve, the validity range $Min(F)\le F\le Max(F)$ for each curve fitting and the root means square (RMS) are provided. Note that for each antenna width, there are ten resonance equations and curves; each curve is labeled by a number shown in Table 2 and Fig. 7.

**Table 2:** Peano_1. Parameters for the resonance frequency equations, feeding point placed at 0.125 (1/8) of the length *S*, for three different antenna widths.

Curve	Peano iteration 1 d = 0.25 mm					Peano iteration 1 d = 0.50 mm					Peano iteration 1 d = 1.00 mm				
	a	b	RMS	Min(F)	Max(F)	a	b	RMS	Min(F)	Max(F)	a	b	RMS	Min(F)	Max(F)
	[m MHz]	[$\times 10^{-5}$ m ]	[$\times 10^{-7}$]	[MHz]	[MHz]	[m MHz]	[$\times 10^{-5} m$]	[$\times 10^{-7}$]	[MHz]	[MHz]	[m MHz]	[$\times 10^{-5} m$]	[$\times 10^{-7}$]	[MHz]	[MHz]
0	39.3092	61.0359	2292.47	390.3627	2668.6079	39.4893	90.5418	2711.02	392.2852	2686.0016	39.7111	142.6563	3320.47	398.4707	2897.3903
1	44.5263	46.9969	1570.46	441.6358	3000.2113	44.6579	73.6020	1593.60	443.0056	2654.1517	44.8145	122.2558	1824.30	448.9260	2827.6120
2	74.3279	4.3839	338.23	734.4907	2950.3644	74.2489	17.3887	415.53	732.8566	2933.6087	74.1533	45.2071	460.92	737.5908	2795.0774
3	85.6626	4.1398	205.87	846.4442	2933.6490	85.5866	15.7786	340.64	844.6097	2918.2492	85.4576	44.2684	575.10	849.9753	2995.0108
4	152.3098	53.4593	134.15	1512.2041	2888.9719	152.5458	90.2732	134.15	1516.3244	2899.9560	152.7063	168.5865	34.10	1537.5967	2976.0295
5	172.5254	− 12.2380	18.02	1701.9094	2905.8590	172.4732	− 21.0105	15.19	1695.7440	2988.6418	172.5326	− 45.0220	100.71	1700.6099	2901.8698
6	188.3153	25.2616	15.28	1864.5450	2989.2240	188.3746	46.8244	13.77	1864.4980	2988.5377	188.4135	91.1013	2.61	1882.4571	2939.8708
7	220.0766	18.2876	45.99	2177.4833	2931.6419	220.7049	0.9298	55.73	2174.5757	2923.4956	221.4859	− 42.3829	67.98	2183.6977	2936.4188
8	230.9235	119.4539	16.02	2307.9361	2958.4236	230.8036	241.5736	59.59	2329.4279	2994.3050	209.1765	1453.8268	0.00	2419.2963	2476.5837
9	265.3432	− 145.7664	13.65	2583.4986	2991.2341	264.9992	− 269.3931	10.73	2543.3142	2938.0839	299.8693	− 2032.6858	0.00	2471.5819	2513.0073

**Table 3:** Peano_1. Parameters for the resonance frequency equations, feeding point placed at 0.3125 (or 5/16) times the length *S*, for three different antenna widths.

Curve	Peano iteration 1 d = 0.25 mm					Peano iteration 1 d = 0.50 mm					Peano iteration 1 d = 1.00 mm				
	a	b	RMS	Min(F)	Max(F)	a	b	RMS	Min(F)	Max(F)	a	b	RMS	Min(F)	Max(F)
	[m MHz]	[$\times 10^{-5} m$]	[$\times 10^{-7}$]	[MHz]	[MHz]	[m MHz]	[$\times 10^{-5} m$]	[$\times 10^{-7}$]	[MHz]	[MHz]	[m MHz]	[$\times 10^{-5} m$]	[$\times 10^{-7}$]	[MHz]	[MHz]
0	39.2984	60.2803	2263.68	390.2295	2666.7312	39.4761	89.4420	2675.07	392.1154	2683.3688	39.6948	140.9701	3270.51	398.2441	2893.1110
1	55.1491	19.2913	144.22	545.6984	2893.2110	55.1587	29.2798	40.89	545.0028	2872.0267	55.1581	66.5846	4979.25	550.1723	2999.2615
2	74.2841	2.8732	374.15	733.9553	2947.0243	74.1964	15.3328	462.03	732.1994	2929.3659	74.0896	42.3135	520.57	736.7560	2789.9076
3	109.6878	− 93.3115	1327.77	1073.8799	2876.7740	108.8322	− 107.8558	2015.46	1061.6008	2982.0320	107.6416	− 119.5877	4209.90	1053.6961	2987.5292
4	152.2292	59.2135	129.48	1512.2698	2890.6114	152.3810	108.8859	33.21	1517.5458	2907.4338	151.8770	244.1790	3221.91	1540.8696	2783.5914
5	163.7704	− 21.0098	63.88	1614.1744	2953.0878	163.7012	− 46.7480	27.29	1605.4240	2924.8411	164.2686	− 115.3645	6879.40	1609.5107	2827.5240
6	188.5018	28.6246	24.92	1867.0054	2993.7604	188.6072	50.8967	28.07	1867.5426	2994.1309	188.7243	94.4362	84.30	1886.1424	2946.1252
7	222.1877	− 29.5786	9.87	2188.0637	2941.1202	221.8565	− 45.9104	47.33	2175.9801	2999.8135	221.2642	− 55.5042	750.31	2179.2406	2928.1693

##### Example 3

shows how the Peano_1 antenna produces three different equations of the type $L=aF^{-1} +b$, each with a validity frequency range and RMS, for a particular antenna width (0.25 mm, 0.5 mm, and 1.0 mm). The parameters *a* and *b* are obtained from Table 2; this is done for the feeding point 1/8 times *S*. From Table 2, the resonance curve labeled 0, for *d*=0.25 mm, we obtain the following equation, $L=39.3092F^{-1}+ 61.0359\times 10^{-5}$, for the resonance frequency *F* and the distance *L*, valid in the frequency range $390.3627MHz\le F\le 2668.6079 MHz$, with a RMS=$2292.47\times 10^{-4}$. If we change to *d*=0.5mm, then the new equation is $L=39.4893F^{-1}+ 90.5418 \times 10^{-5}$, valid for the resonance frequency range of $392.2852 MHz\le F\le 2686.0016 MHz$ with RMS equal to $2711.02\times 10^{-7}$. Finally, if we change to *d*=1mm, the equation obtained has the form $L=39.7111F^{-1}+ 142.6563 \times 10^{-5}$, for the resonance frequency validity range of $398.4707MHz\le F\le 2897.3903 MHz$, with a *RMS*=$3320.47\times 10^{-7}$. These curves are graphically represented in Fig. 5 with green + symbols and in Fig. 7 with a blue ($\circ$) line.

In Table 3 we show the parameters of the resonance frequency curves $L=aF^{-1}+b$ for the Peano_1 antennas in three different antenna widths *d* (0.25 mm, 0.5 mm, and 1 mm), with the feeding point placed at 0.3125 (or 5/16) times the antenna length *S*. Note that eight resonance curves exist instead of the ten resonance frequency curves if the feeding point is placed at 0.125 times *S* for the Peano_1 antenna simulation.

**Table 4:** Peano_1. Parameters for the resonance frequency equations, feeding point placed at 0.4375 (7/16) of the length *S*, for three different antenna widths.

Curve	Peano iteration 1 d = 0.25 mm					Peano iteration 1 d = 0.50 mm					Peano iteration 1 d = 1.00 mm				
	a	b	RMS	Min(F)	Max(F)	a	b	RMS	Min(F)	Max(F)	a	b	RMS	Min(F)	Max(F)
	[m MHz]	[$\times 10^{-5} m$]	[$\times 10^{-7}$]	[MHz]	[MHz]	[m MHz]	[$\times 10^{-5} m$]	[$\times 10^{-7}$]	[MHz]	[MHz]	[m MHz]	[$\times 10^{-5} m$]	[$\times 10^{-7}$]	[MHz]	[MHz]
0	39.2973	60.1865	2260.95	390.2147	2666.4921	39.4748	89.2827	2670.79	392.0961	2683.0132	39.6932	140.7051	3268.41	398.2179	2892.4043
1	66.8128	6.0734	258.24	660.3189	2882.5796	66.7536	7.9394	475.26	658.2646	2852.6715	66.6550	38.9303	7085.54	663.3393	2939.2161
2	74.2708	2.5174	387.44	733.8011	2946.1433	74.1800	15.0163	477.94	732.0183	2928.4276	74.0705	41.9207	531.10	736.5356	2788.8789
3	85.4541	− 17.1844	490.79	842.6752	2906.4613	85.2802	− 4.0586	784.99	840.0448	2890.0757	84.9738	9.2774	7614.70	841.3910	2933.3147
4	151.9124	61.2614	109.78	1509.4419	2999.7396	152.0812	109.2142	64.27	1514.5937	2901.7609	151.6684	245.6978	2999.43	1539.9508	2884.1398
5	171.3972	− 61.6635	113.09	1682.6356	2962.1881	171.0650	− 85.2927	131.60	1671.4105	2931.8296	171.3063	− 199.9093	10051.61	1660.0625	2888.8492
6	188.5107	43.1025	15.55	1869.7816	3000.8257	188.4165	92.3221	80.57	1873.4121	2917.9108	186.7243	274.7258	3953.39	1899.6371	2658.9099
7	198.9010	− 34.8369	48.43	1957.7584	2942.4918	198.7195	− 71.0374	10.67	1944.2246	2913.3032	199.4387	− 167.7493	7788.20	1945.8511	2670.5862
8	232.0882	108.5704	34.83	2317.0356	2969.1677	232.7732	178.4546	29.43	2334.3401	2995.1421	232.6510	360.3901	937.80	2389.2274	2929.4060
9	259.6622	− 34.6057	1.63	2555.8315	2964.3166	259.5831	− 53.5902	0.26	2544.0365	2948.6041	258.8893	− 99.6757	3333.82	2536.3259	2944.5777

**Table 5:** Peanno_1. Parameters for the VSWR<2 equations, *d* equal to 0.25 mm, 0.5 mm, and 1.0 mm.

		Peano iteration 1 d = 0.25 mm					Peano iteration 1 d = 0.50 mm					Peano iteration 1 d = 1.00 mm				
feed	curve	a	b	RMS	Min(F)	Max(F)	a	b	RMS	Min(F)	Max(F)	a	b	RMS	Min(F)	Max(F)
		[m MHz]	[$\times 10^{-5}m$]	[$\times 10^{-7}$]	[MHz]	[MHz]	[m MHz]	[$\times 10^{-5}m$]	[$\times 10^{-7}$]	[MHz]	[MHz]		[$\times 10^{-5}m$]	[$\times 10^{-7}$]	[MHz]	[MHz]
1/8 S	0	39.3205	60.3841	2305.10	390.3750	2668.6250	39.5027	89.7354	2631.12	392.3750	2686.2500	39.7195	142.4375	3257.86	398.5000	2898.3750
	1	74.3302	4.0267	411.62	734.5000	2950.2500	74.2484	17.3755	519.44	732.8750	2933.7500	74.1472	46.1878	542.02	737.6250	2796.0000
5/16 S	0	74.3252	3.0872	496.59	734.3750	2949.1250	74.2390	16.3716	451.96	732.6250	2932.1250	74.1412	43.8112	598.00	737.3750	2793.7500

The feeding point is placed at 0.125 (or 1/8) and 0.3125 (5/16) times the length *S*.

Table 4 shows the parameters of the resonance frequency curves $L=aF^{-1}+b$ for the Peano_1 antennas with three different antenna widths (0.25mm, 0.5mm, 1 mm), and feeding point placed at 0.4375 (or 7/16) times the length of the antenna *S*. In this case, there are ten resonance curves. The Peano_1 antenna parameters, feeding point located at 0.5 (or 1/2) times the length *S* are shown in Table 6. Only two resonance curves are possible for the antenna width of 1mm. Tables 2, 3, 4, and 6 provide the information needed to design, through their resonance frequencies, many Peano_1 antennas, in a similar way.

**Table 6:** Peano_1. Parameters for the resonance frequency equations, feeding point placed at 0.5 (or 1/2) times the length *S*, for three different antenna widths.

Curve	Peano iteration 1 d = 0.25 mm					Peano iteration 1 d = 0.50 mm					Peano iteration 1 d = 1.00 mm				
	a	b	RMS	Min(F)	Max(F)	a	b	RMS	Min(F)	Max(F)	a	b	RMS	Min(F)	Max(F)
	[m MHz]	[$\times 10^{-5}m$]	[$\times 10^{-7}$]	[MHz]	[MHz]	[m MHz]	[$\times 10^{-5}m$]	[$\times 10^{-7}$]	[MHz]	[MHz]	[m MHz]	[$\times 10^{-5}m$]	[$\times 10^{-7}$]	[MHz]	[MHz]
0	39.2972	60.1786	2260.67	390.2134	2666.4728	39.4747	89.2690	2670.49	392.0943	2682.9818	39.6542	147.8141	2036.90	398.2156	1680.1156
1	76.0477	− 3.0599	215.72	750.8984	2788.6502	75.9584	3.4937	672.08	748.7391	2986.3350	75.8239	21.3563	457.06	752.4315	1693.7680
2	117.7611	450.3067	5674.08	1218.6418	2655.2450	103.2134	2078.9249	516.22	1279.1509	1345.8464					
3	150.2310	− 517.9883	7616.95	1409.4736	2733.5770	183.4192	− 3575.2250	918.78	1336.0216	1376.0724					
4	188.4530	28.9678	21.46	1866.5882	2993.1564	188.5393	52.5854	21.02	1867.1871	2993.8708					
5	220.6946	− 72.3041	75.68	2164.3177	2983.5970	219.7662	− 92.3438	107.34	2145.7648	2953.1257					
6	270.0757	145.9924	2.14	2706.4349	2942.3151	269.5047	311.0432	15.54	2739.1819	2981.6192					
7	299.2476	− 107.8169	0.00	2924.3910	2982.6875	300.1823	− 251.8570	0.00	2885.8529	2942.4280

**Table 7:** Peano_1. Polynomial coefficients needed to calculate the bandwidth around the central frequency such that VSWR< 2. For antenna width of 0.25 mm, 0.5 mm, and 1.0 mm, feeding point placed at 0.125 (or 1/8) and 0.3125 (or 5/16) times the length *S*.

Feed	Curve	Peano iteration 1 d = 0.25 mm						Peano iteration 1 d = 0.50 mm						Peano iteration 1 d = 1.00 mm					
		$\alpha$	$\beta$	$\gamma$	RMS	Min(F)	Max(F)	$\alpha$	$\beta$	$\gamma$	RMS	Min(F)	Max(F)	$\alpha$	$\beta$	$\gamma$	RMS	Min(F)	Max(F)
		[$\times 10^{-8}$]	[$\times 10^{-5}$]			[MHz]	[MHz]	[$\times 10^{-8}$]	[$\times 10^{-5}$]			[MHz]	[MHz]	[$\times 10^{-8}$]	[$\times 10^{-5}$]			[MHz]	[MHz]
1/8 S	0	− 5.5556	36.3870	0.8283	0.1138	390.38	2668.62	− 6.3695	45.3588	0.9312	0.1160	392.38	2686.25	− 7.1773	59.2836	1.0448	0.1279	398.50	2898.38
	1	− 4.9990	40.1808	1.3894	0.0602	734.50	2950.25	− 4.8191	46.4522	1.5673	0.0473	732.88	2933.75	− 7.0395	65.3624	1.7171	0.0612	737.62	2796.00
5/16S	0	− 5.2276	41.1223	1.6628	0.0511	734.38	2949.12	− 5.8774	49.5699	1.8295	0.0499	732.62	2932.12	− 9.0847	68.7123	1.9768	0.0673	737.38	2793.75

Despite the Peano_1 antenna relative simplicity, we observe some intriguing patterns in the resonance frequency curves for antennas with feeding points placed at (1/8, 5/16, 7/16, 1/2, 9/16, 11/16, 7/8) times the length *S*, and the simulated *d*: 0.25mm, 0.5mm, and 1mm. The number of resonance curves and their parameter values vary for antennas with the same feeding point but with different microstrip Peano antenna widths or the other way around. Concerning these resonance curves, the Peano_1 antennas with feeding points placed at 1/8 times *S* are described in Fig. 5; there are similar curves for the three different antenna widths. However, it is also necessary to note that there are few curves for some antenna widths and feeding points location.

The computing time needed to calculate the Peano_1 antenna properties with three different widths (0.25 mm, 0.50 mm, and 1 mm) for the hardware used in this research is as follows. The antennas with the feeding point at 1/8 times *S*; 284.3517 h. The antennas with the feeding point at 5/16 times *S*, 233.6662 h. The antennas with the feeding point placed at 7/16 times *S*, 210.0715 h. Finally, the antennas with the feeding point placed at 1/2 times *S*, 196.3736 h.

#### Peano_2 and Peano_3

For higher iterations of the production rule *i*, in particular, *i* = 2,3, the radiation properties, particularly the resonance frequency of Peano antennas, can be obtained by using the Peano_antennas application available in the reference^34^. However, for $i>3$ the application only provides the geometry of the Peano antenna so far.

### Peano antennas: Equations for the frequencies such that VSWR< 2 

We report the Peano antenna radiation features for a frequency range (also called bandwidth *BW*) such that VSWR< 2, which is a needed condition for a suitable antenna performance^4^. At first, the mean value of this frequency range is obtained and called the central frequency *f*. GPlearn shows that *f* can be related to *L* by equations of the form $L=af^{-1}+b$, with the parameters *a* and *b* from Table 5 for Peano_1 antennas with feeding point placed at 0.125 (or 1/8) and 0.3125 (or 5/16) times the length *S*. The bandwidth *BW* (or frequency range) is reported as a percentage of the central frequency *f* by using a second-degree polynomial fitting; namely by equations of the form *BW*=$\alpha f^{2}+\beta f+\gamma$, where the parameters $\alpha$, $\beta$, and $\gamma$ are taken from Table 7 for Peano_1 and feeding point placed at 0.125 (or 1/8) and 0.3125 (or 5/16) times the length *S*.

#### Peano_1

**Figure 8:**
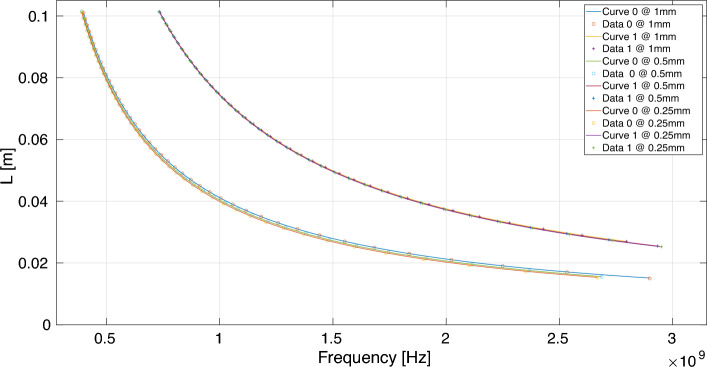
Peano_1 feeding point placed at 0.125 (or 1/8) times *S*, VSWR< 2 curves for the following antenna widths: (**a**) 0.25 mm, (**b**) 0.5 mm, and (**c**)1.0 mm.

Figure 8 shows the simulated data for the frequency *f* and *L* grouped in curves, which correspond to equations of the form $L=af^{-1}+b$ (obtained by GPlearn), for the feeding point placed at 0.125 (1/8) times the length *S* and three different antenna widths: 0.25 mm, 0.5 mm , and 1.0 mm.

Table 5 shows the parameters of the equations $L=af^{-1}+b$ that govern the curves in Fig. 8 for the antennas with feeding point placed at 1/8 and 5/16 times *S*. The numbers in these curves correspond to the labels in the Table. Here, we present the simulated values for three different antenna widths: 0.25 mm, 0.5 mm, and 1.0 mm.

##### Example 4

Let us consider the curve labeled as zero in Fig. 8 and Table 5 with a feeding point placed at 0.125 (or 1/8) times *S*, and width equal to 0.25mm; the equation $L=39.3205f^{-1}+60.3841 \times 10 ^{-7}$ relates the central frequency *f* with the length *L* for electrically efficient antennas. This equation has an RMS of 2305.10 $\times 10^{-7}$, and it is valid in the range $390.3750\,MHz\le f\le 2668.6250MHz$. The same antenna but with a width *d* of 0.5mm would follow the equation $L=39.5027f^{-1}+89.7354 \times 10 ^{-7}$, valid in the range $392.3750MHz \le f \le 2686.2500$, RMS equal to $2631.12\times 10^{-7}$. Finally, if the antenna width is 1.0mm, then the equation that governs the suitable antennas is $L= 39.7195f^{-1}+142.4375 \times 10 ^{-7}$, valid in $398.5000MHz\le f\le 2898.3750MHz$ with an RMS of $3257.86 \times 10^{-7}$. There are no Peano_1 antennas with feeding points placed at 7/16 times *S* and 1/2 times *S*, so the VSWR values are smaller than 2.

We complete this Peano_1 antenna VSWR description by calculating the bandwidth *BW* around the central frequency where VSWR< 2. Namely, we search for the upper and lower bound of *BW* as a percentage of the central frequency; the percentage values are related to the center frequency using a second-degree polynomial fit $BW=\alpha f^{2}+\beta f+\gamma$. Please, find in Table 7 the coefficients of the resulting bandwidth equations $\alpha$, $\beta$, $\gamma$ calculated for antenna widths of 0.25mm, 0.5mm, and 1mm. Besides, these coefficients are given for feeding points placed at 0.125 (1/8) and 0.3125 (5/16) times the length *S*.

##### Example 5

For a central frequency of 1000 MHz, we can simulate the suitable Peanno_1 antennas with VSWR < 2 and the corresponding bandwidth around the central frequency. From Table 5 any of the three different microstrip Peano_1 antenna widths (0.25 mm, 0.5 mm, 1.0 mm) and two feeding points (1/8 and 5/16 times *S*), can be chosen; since 1000 MHz is, in each case, a valid frequency range. It is easy to see that we obtain a suitable design if the feeding point is placed at 1/8 times *S* with an antenna width of 0.25mm. In these conditions, the equation to determine the size *L* of the antenna is $L=39.3205f^{- 1}+60.3841\times 10^{-5}$; the *L* value for 1000 MHz from this equation is $3.9924 \times 10^{-2}m$, RMS equals to $2305.10 \times 10^{-7}$. Table 7 shows the parameters of the polynomial that provide the bandwidth values around the central frequency of 1000MHz; thus, $BW =-5.5556\times 10^{-8}f^{2}+36.3870\times 10^{-5}f+0.8283$ is obtained, with an RMS of 0.1138 that for 1000MHz gives $BW=1.1366\%$. To summarize, the characteristics of the proposed Peanno_1 antenna with a VSWR< 2 are: L=3.9924cm, d=0.25mm, feeding point supply at 1/8 times *S*, and BW =1.1366%.

#### Peano_2 and Peano_3

**Figure 9:**
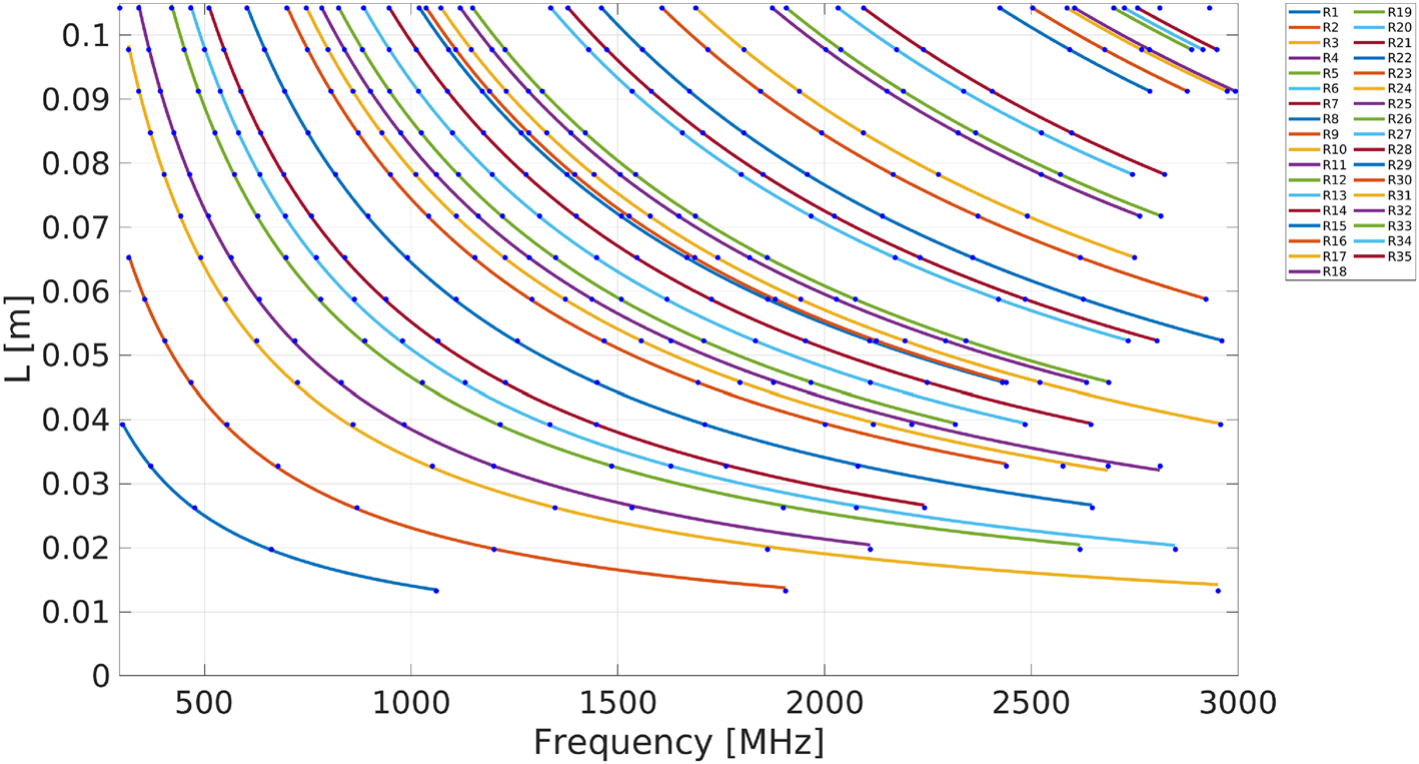
Frequency resonance curves of a Peano antenna iteration 3 with the feed point at 1/2S and a width of 0.5 mm, the blue points correspond to the data used to adjust the functions in the Peano_antennas application.

For higher iterations of the production rule *i*, in particular, *i*=2,3, the radiation properties, in particular, the frequencies such that VSWR<2 of Peano antennas, can be obtained by using the Peano_antennas application available in the reference^34^. However, for $i>3$, the application only provides the geometry of the Peano antenna so far. For instance, Fig. 9 shows the Peano iteration 3 frequency resonances. The equations that describe these curves are in the Peano_antennas application; the blue dots in Fig. 9 are the data obtained from the computational simulations.

**Figure 10:**
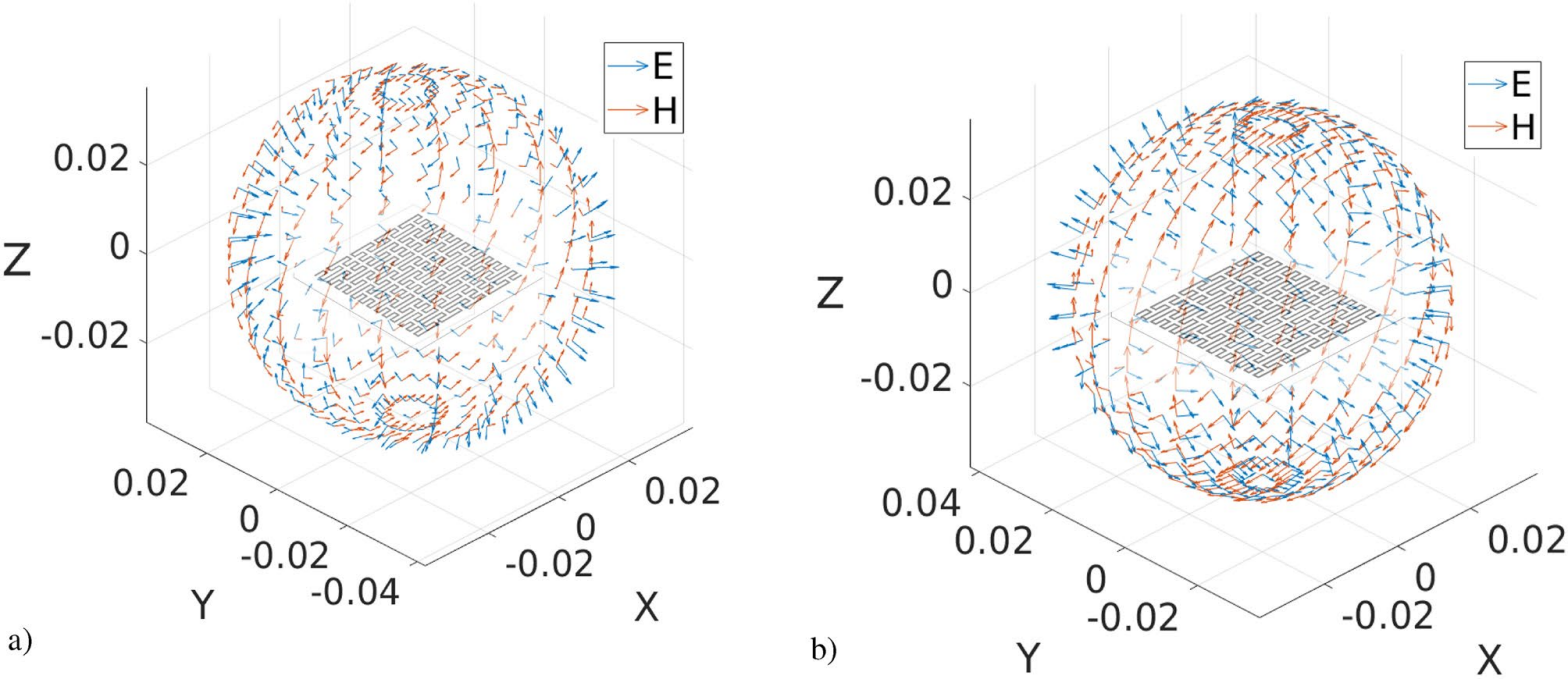
A Peano iteration 3 antenna in three-dimensional space: the electric (E) and magnetic (H) fields generated at a resonance frequency of (**a**) 476,4531 MHz and (**b**) 2241,8604 MHz which simultaneously has a VSWR of 1,1102. This Peano iteration 3 metallic antenna has air as a substrate; the feeding point is placed at 0.5*S*, *d* = 0.25, and *L* = 5.25 cm.

Figure 10 shows the magnetic (H) and electric (E) fields of a Peano fractal antenna iteration 3 at a resonance frequency of (a) 476,4532 MHZ and (b) 2241,8604 MHz, where the antenna resonates and has a VSWR equal to 1,1102 simultaneously. For the calculation of resonance frequencies, we use the bisection method such that each new point around the zero crossing of the reactance is calculated in each step of the method.

The bisection method is based on the intermediate value theorem, which establishes that if a function is continuous in a closed interval [A, B] and takes opposite values at the ends, then the function has at least one zero in this interval. The bisection method works by dividing the interval [A, B] in half and determining which subinterval the function zero is; we repeat this process until a desired precision is reached or until the iterations are exhausted. A more complete and detailed simulation of the Fig. 10 antenna has been carried out; its directivity can be seen from 100 MHz to 15 GHz in^38^ https://youtu.be/62ZfwXYbX3w; and the E and H fields for the same frequency range in^39^ https://youtu.be/YB1539T0pCw.

## Conclusions

In this paper, we produce the geometry of the FASS antennas following the Peano curve. We set the iteration of the production rules equal to 1,2,3. Then, the MoM numerical method solves the Maxwell equations over the Peano_i metallic antenna (the Peano antenna produced by the iteration *i*) with air as a substrate.

Let us choose $i=$1, 2, 3, the microstrip antenna width *d* from the set (0.25 mm, 0.5 mm, 1.0 mm), $L\le$.10m, the frequency range from 290 MHz to 3100 MHz (including the UHF band), and the feeding point from the set (1/8, 5/16, 7/16, 1/2, 9/16, 11/16, 7/8) times the length *S*. Then, our model delivers the main antenna electromagnetic properties directly, the patterns these data follow, and the corresponding governing equations.

Using genetic programming (GPlearn) as a novel tool, we find the best equations to fit the following Peano antenna radiation properties; (a) the resonance frequencies and (b) the central frequencies such that VSWR < 2, both as functions of *L*. For (b), we report the bandwidth *BW* around the central frequencies such that VSWR < 2 as a percentage of the central frequency. We conjecture that the existence of these curves is guaranteed for other iterations and parameters. Thus, we provide the systematic way and data needed to simulate efficient Peano antennas; our approach can be extended easily to include additional cases in the Peano_antennas application. The simulations provided in this paper give some insight into the technological applications of the patterns found in the Peano_i antenna resonance frequencies and VSWR<2. These calculations are presented within the UHF band; analysis of cases beyond this band is not in this paper’s scope.

The simulations performed, plus the symbolic regressions (via GPlearn), produce the mathematical relation $L=aF^{-1}+b$ that best describes the resonance frequency as functions of *L* and the VSWR<2 central frequency relation with *L*. We look for the crossing by zero for the first case and provide numerical methods for the best values of parameters a and b. For the second case, we look for all the values smaller than 2 (frequency range) and again fit the $L=af^{-1}+b$ through numerical methods and GPlearn. Here *f* is the central frequency within the frequency range; finally, we obtain the bandwidth around the central frequency as a percentage. For both cases, we optimize our numerics by the least possible RMS.

We observe that the number of resonance frequency curves increases with increasing (production rules) iterations of the Peano antenna. Additionally, the change in antenna width, even by half a millimeter, favors the change in the number of resonance curves, which must also be considered for the design and applications of the antennas presented here.

From Fig. 4 and the corresponding Figures for Peanno_2 and Peanno_3 available in Peanno_antennas^34^, we can see the unmistakable patterns in the graphs feeding points vs. frequency for the; (a) logarithm of the resistance, (b) resonance, (c) return loss, and (d) logarithm of the VSWR. Furthermore, we observe how the graphs present patterns that follow increasingly complex curves suitable for being expressed by equations or relations in all these cases. Thus, we obtain equations for resonance frequencies and frequencies such that the VSWR is smaller than two from the data clustered in curves. In our case, we cluster the resonance frequency and frequencies such that VSWR < 2 in curves described by equations retrieved using GPlearn, as a successful first approach that supports our observations of patterns over these Figures; this is undoubtedly the best achievement reported in this paper. Besides, figures like Fig. 4 might be used as a hallmark of the Peano antenna for other purposes.

Analyzing the Peano antennas reported in this paper and produced in the Peano_antennas application, we conclude that the FASS geometry induces patterns in the radiation properties of the antennas suitable for being approached by equations whose parameters can be deduced from the geometry.

## Data Availability

All data used in this paper, plus additional cases, are included in the following GitHub address https://github.com/moroyoqui-UNAM/PEANOANTENNAS.
